# Blood biomarkers of long COVID: A cross-sectional evaluation of key cytokines and the coordination of inflammation

**DOI:** 10.1371/journal.pone.0360525

**Published:** 2026-10-21

**Authors:** Callum Thomas, Ruth E. Ashton, Etheresia Pretorius, Rebecca Owen, Massimo Nunes, Jan H. Pretorius, Isaac T. Shawa, Corinna Chidley, Mark A. Faghy

**Affiliations:** 1 School of Health, Sport and Rehabilitation, College of Health and Humanities, University of Derby, Derby, United Kingdom; 2 Research Centre for Healthcare and Communities Transformation, Coventry University, Coventry, United Kingdom; 3 Department of Physiological Sciences, Faculty of Science, Stellenbosch University, Stellenbosch, South Africa; 4 Department of Economics, Faculty of Economic and Management Sciences, Stellenbosch University, Stellenbosch, South Africa; 5 School of Sport, Exercise and Health Sciences, Loughborough University, Loughborough, United Kingdom; Noorda College of Osteopathic Medicine, UNITED STATES OF AMERICA

## Abstract

**Introduction:**

Long COVID is a complex pathophysiological condition linked to a heterogenous symptom profile. Dysregulation of 1) immunological and inflammatory responses, 2) endothelial/vascular function, and 3) metabolic and clotting processes have been identified; however, there remains a lack of clinically validated diagnostic biomarkers. The study aim was to investigate dysregulation of cytokines, chemokines and growth factors in confirmed Long COVID cases grouped by disease severity.

**Methods:**

One-hundred-and-twenty-seven participants provided venous blood samples for cytokine profiling utilising Luminex assays. Participants were grouped into those with no knowledge of SARS-CoV-2 infection (n = 21), acute infection within 3-months (n = 22), spontaneous resolvers (>3-months, n = 21) and those with confirmed Long COVID (n = 63). Long COVID severity was grouped according to the post-COVID-19 functional status (PCFS) and defined as those with negligible (4A; n = 9), mild (4B; n = 20), moderate (4C; n = 24), and severe limitations (4D; n = 10). Cytokines were assessed for normality using Shapiro-Wilk tests and differences measured between groups using Kruskal-Wallis tests. Pairwise correlation and principal component analysis were performed.

**Results:**

Interleukin-12 (p40/p70) was 22% greater in the Long COVID group (Median [IQR]: 58 [49] pg/mL, p = 0.046) and 30% greater in the acutely infected group (64 [36] pg/mL; p = 0.039) against non-infected controls (45 [25] pg/mL). Following PCFS classification, fibroblast growth factor-basic was 25% lower in Long COVID 4B versus spontaneous resolvers (24 [7] vs. 32 [24] pg/mL; p = 0.025). Pairwise correlation differences demonstrated that three Long COVID subgroups had strong, positive associations between cytokines when compared to non-infected controls; however, PCA was unable to determine any coordinated cytokine variation that was group specific.

**Discussion and conclusions:**

Elevated interleukin-12 (p40/p70) and reduced basic fibroblast growth factor suggest persistent immune activation and possible subgroup-specific Long COVID; however, these findings were marginally significant and exploratory. A coordinated but partial immunological/inflammatory profile may exist in some of those with Long COVID, suggesting a rationale for subgrouping upon a biological, rather than self-report basis but larger longitudinal studies are required to confirm this hypothesis.

## Introduction

Long COVID is defined as the persistence and emergence of new symptoms following a Severe acute respiratory syndrome coronavirus 2 (SARS-CoV-2) infection that has a deleterious impact on daily life and functional status affecting children, adolescents, and adults [1]. Previous reviews including our group have evaluated the biomarkers of Long COVID, with a range of 113–525 individual markers being identified [2–4]; however, there remains no diagnostic biomarkers for Long COVID. Diagnostic biomarkers are important not only to identify those living with Long COVID but to elucidate the underlying pathophysiological mechanisms which may vary from patient-to-patient, monitor disease progression, and understand the impact of management and treatment strategies [5].

Key themes established from previous reviews suggest that no single biomarker is likely to identify individuals living with Long COVID and rather a profile that is reflective of a clinical symptomatology is required. A review by Espin *et al.* [2] classified symptom domains with candidate blood biomarkers; for example, pulmonary/respiratory Long COVID (e.g., CD4+ and CD8 + T-cell subsets), neurological Long COVID (e.g., interleukin-6 [IL-6] and tumor necrosis factor-alpha [TNF-α]), and gastrointestinal Long COVID (e.g., autoantibody La/SS-B). Previous work that adopted the World Health Organisation (WHO) definition of Long COVID [6] and evaluated the blood biomarkers of Long COVID reported symptom(s) aligning with candidate blood biomarkers, but no definitive conclusions could be drawn [4]. This aligns with the wide variety of symptoms reported from patients which undulate and are prone to exacerbation [7–9], and this underlines the difficulty and ambiguity of understanding symptoms alongside biomarkers. Instead, it seems biomarker domains align well with disturbances in (1) immunological and inflammatory dysfunction, (2) endothelial/vascular dysfunction and (3) metabolic and clotting abnormalities (4).

A key limitation identified in previous research is the inconsistent definition of Long COVID used in inclusion criteria, along with insufficiently detailed comparator groups. Our groups review, for instance, excluded nearly 200 studies due to challenges with Long COVID definitions and comparator group specifications [4]. By including well-defined Long COVID and comparator groups, together with previous medical history and current comorbidities, it is anticipated that diagnostic biomarkers could be found from this recruitment approach. Comparator groups provide valuable data that are representative of specific populations as opposed to clinical ranges that are subject to statistical and biological variability, small reference populations, and often lack context [10]. Whilst blood biomarkers may have been recognised from some questionnaires (e.g., cognitive impairment in people with Long COVID has been associated with elevated interferon-gamma (IFN-γ; [11]) many of the tools are compartmentalised and do not account for the severity of functional limitations on everyday life. By contrast, the post-COVID-19 Functional Status (PCFS; [12]) scale offers a useful and clinically applicable assessment tool to group people living with Long COVID as opposed to symptom specific scales. The PCFS encapsulates the broad symptom profile and functional impact that Long COVID has on the everyday lives of patients and, to the best of the authors’ knowledge, only one study has implemented the PCFS to stratify people living with Long COVID defined by the WHO alongside blood biomarker profiling [13]. Through acute COVID-19 progression & PCFS classification, this study observed increased alanine aminotransferase in the more functional PCFS group with mild acute disease, and increased aspartate aminotransferase for the less functional PCFS group with mild acute disease. However, these findings are not contextualised against any comparator groups and the PCFS was used to classify people living with Long COVID into two groups only.

Accordingly, we hypothesized that commonly associated blood biomarkers of inflammation, immunological function, and vascular growth and repair would distinguish those with a confirmed Long COVID diagnosis from comparator groups comprising individuals who had never been diagnosed with SARS-CoV-2 infection or those who had previously been infected but had recovered (spontaneous resolvers). We also anticipated that these blood biomarkers would help distinguish between those who had greater functional Long COVID limitations and those whose limitations were minimal, and between the Long COVID and acutely infected groups. The overall objectives of the study were 1) describe the role of candidate Long COVID blood biomarkers inclusive of suitably detailed comparator groups; 2), determine if perceived severity of Long COVID assessed using the PCFS scale is reflected through blood biomarker profiling.

## Materials and methods

### Participants

Following institutional ethics approval from the University of Derby (ETH2223−0418), 127 participants provided written informed consent and were recruited between May 2023 and April 2024 as part of a single-centre, cross-sectional study. Due to the exploratory nature of our analysis, no formal sample size calculations were made during the study design; however, a target of twenty participants per comparator group and Long COVID subgroup was set based upon the available literature at the time and the minimum group size numbers reported.

Participants were recruited to the study via convenience sampling methods, supported by a social media campaign, word of mouth, and an existing database of Long COVID participants held by the host University. Eligibility was assessed via telephone consultation or email and included adults aged 18 years old and above. People living with Long COVID were included based on meeting the WHO definition for Long COVID [6]. People living with Long COVID were stratified into four groups based on their perceived severity of symptoms using the PCFS with a full description for each included in S1 Table [12]. Three comparator groups consisted of:

1. Uninfected controls (participants were excluded if they suspected they had been infected with COVID-19).
2. Recently SARS-CoV-2 infected individuals (within 3-months of a confirmed polymerase chain reaction [PCR] or lateral flow test).
3. Participants who had recovered from SARS-CoV-2 infection (spontaneous resolvers; beyond 3-months from confirmed PCR or lateral flow test).

Exclusion criteria were, < 18 years of age, any vaccination in the past two months, or any blood samples taken in the past month. Individuals were also excluded if they were feeling unwell or had any known comorbidities or health conditions (e.g., inflammatory arthritis, autoimmune conditions, chronic obstructive pulmonary disease, etc.) that would interfere with sample analysis (with the exception of Long COVID participants). Sufficient English language comprehension and cognitive ability to understand the study protocol, give informed consent and follow instructions were required for participation in this study. Additional eligibility criteria for individuals with Long COVID, as well as comparator groups two and three, included a confirmed COVID-19 diagnosis verified by a lateral flow test, polymerase chain reaction (PCR) test or retrospective confirmation of SARS-CoV-2 infection by a healthcare professional.

### Patient reported outcome measures (PROMs)

PROMs were administered via paper form and delivered by researchers in the face-to-face baseline assessment. The PCFS scale evaluated the ultimate consequences of COVID-19 on functional status and supplements other instruments that measure quality of life, tiredness, or dyspnoea in the acute phase. The PCFS covers the full spectrum of functional outcomes and focuses on both limitations in usual duties/activities and changes in lifestyle in six scale grades, four of which were used to categorise the Long COVID cohort. Symptom profile was also measured to capture: 1) retrospective assessment by the patient of their symptom status at symptom onset (essentially their baseline symptoms); and 2) their current symptom status at the point of testing. The impact of current symptom status on daily life on a scale of 0–10 was also measured.

### Sample collection

Venous blood samples were collected into 5.0 mL serum-separating tube vacutainers from participants at a one-time point visit to the University of Derby to measure cytokine levels. Serum separation was performed within 1–2 hours of collection by centrifuging the blood samples at 2,500 rpm for 10 minutes at room temperature. Serum was aliquoted and stored at −80°C until analysis.

### Cytokine panel analysis

Inflammatory, vascular injury, and immunological cytokines were measured from serum samples using a cytokine 30-plex human panel from Thermo Fisher Scientific (catalogue number: LHC6003M), designed for Luminex™ platforms. This experiment was performed on the Flexmap 3D platform (software: xPONENT 4.2). This panel measured commonly associated Long COVID blood biomarkers which included various interleukins, both IFN-α and IFN-γ, vascular endothelial growth factor-A (VEGF-A), and TNF-α. All analyses followed the manufacturer’s guidelines, and cytokine concentrations were compared across groups to assess changes in systemic inflammation. The laboratory staff were blinded to the severity or clinical details of the participants in this project.

### Data handling

Cytokine data values that were outside the range of detection (OOR) for this array were excluded from the analysis. Values that were above or below the upper or lower limit of quantification (ULOQ/LLOQ) were included in this analysis to preserve biological validity. Many clinically important cytokines in this array had concentrations below the assay’s LLOQ and removing these values could have biased results toward higher concentrations which would misrepresent the dataset.

### Statistical analysis

Data are presented as median (interquartile range; IQR) along with minimum and maximum values or mean ± standard deviation in the case where normality was assumed. Normal distribution was assessed using Shapiro-Wilks tests. Nonparametric data were assessed for all data using Kruskal-Wallis tests with Dunn’s corrections applied to adjust for multiple comparisons in GraphPad Prism (Version 10) with a set α level of 0.05.

#### Correlational analysis.

Data were classified into seven mutually exclusive groups: Non-infected control, Acute, Spontaneous resolvers, and four Long COVID subgroups (4A–4D). Cytokine concentrations were obtained for 19 analytes. Only positive and non-missing readings were retained for analysis. To reduce the influence of skewness and heteroskedasticity, all cytokine concentrations were log-transformed prior to statistical testing and network analysis. When multiple measurements were available per individual–cytokine pair, the median log-transformed value was used to ensure a single observation per subject and analyte.

To examine whether Long COVID alters cytokine co-regulation patterns, group-specific correlation matrices were constructed. For each group, a subject × cytokine matrix of log-transformed concentrations was created. Cytokines with excessive missingness (>10 missing values within a group) were removed. To ensure comparability, analyses were restricted to cytokines present across all groups after filtering.

Spearman rank correlations (ρ) were computed using pairwise complete observations. Spearman correlation was chosen to reduce sensitivity to outliers and avoid distributional assumptions while capturing monotonic relationships. Correlation matrices were visualised using fixed-scale heatmaps (−1 to 1) with identical cytokine ordering across groups. To formally assess differences in cytokine associations, we compared pairwise correlations of the non-infected control group with all other remaining groups. For each cytokine pair, Spearman correlations were calculated in the non-infected control group (r_1_) and the comparison group (r_2_). Correlations were transformed using Fisher’s r-to-z transformation to approximate normality. Differences between groups were evaluated using the standard z-test for independent correlations:


$$\begin{array}{c} Z=\;\frac{z_{\text{1}}-z_{\text{2}}}{\sqrt{\frac{\text{1}}{n_{\text{1}}-\text{3}}+\frac{\text{1}}{n_{\text{2}}-\text{3}}}} \end{array}$$

where $n_{\text{1}}$ and $n_{\text{2}}$ denote group sample sizes. Two-sided p-values were computed assuming asymptotic normality.

To account for multiple testing across cytokine pairs, p-values were adjusted using the Benjamini–Hochberg false discovery rate (FDR) procedure. For each group, we report: 1) the 15 cytokine pairs with the smallest FDR-adjusted p-values, and 2) the 15 statistically significant pairs (FDR < 0.05) with the largest absolute change in correlation (|Δρ|). Correlation change is defined as:


$$\begin{array}{c} \Delta \rho =\rho_{group}-\rho_{control} \end{array}$$

Positive values indicate stronger positive associations relative to non-infected control, whereas negative values indicate attenuated or more negative associations. Differential correlations were visualised using faceted lollipop plots displaying the magnitude and direction of Δρ, with a zero-reference line to facilitate interpretation.

#### Principal component analysis.

To study global cytokine profile structure across groups, we performed principal component analysis (PCA). Given the high dimensionality and correlation among the 19 cytokines, PCA was used to reduce the data to a smaller set of orthogonal components capturing shared variance. This approach allows summarisation of the overall cytokine profile into dominant axes of variation, facilitating group-level comparisons while mitigating multiple testing across individual analytes.

A subject × cytokine matrix was constructed using raw concentration values. Missing values (due to mismeasurement) were addressed using multiple imputation via predictive mean matching (10 imputations, 20 iterations). Prior to imputation, cytokine concentrations were log-transformed (log(x + 1)) to reduce skewness and stabilise variance. Cytokines with zero variance or entirely missing values were excluded from the imputation procedure. One completed dataset was retained for downstream analysis. PCA was then performed on the imputed cytokine matrix using singular value decomposition, with variables standardised to unit variance to account for scale differences across analytes. Principal component scores were extracted for each participant. The proportion of variance explained by each component was calculated from the eigenvalues of the covariance matrix. To assess group separation along the dominant axis of variation, distributions of principal component 1 (PC1) scores were compared between the control group and each non-control group using two-sided Wilcoxon rank-sum tests, with p-values adjusted using the Benjamini–Hochberg procedure.

## Results

### Participants

Four groups were included in the analysis consisting of Long COVID (median [IQR] = 53 [14], range: 30–80 years old), non-infected controls (47 [33], range: 22–76 years old), recently infected individuals (44 [29], range: 23–64 years old), and spontaneous resolvers from SARS-CoV-2 infection (42 [44], range: 21–74 years old). A significant difference in age was found between the Long COVID and spontaneous resolver group (p = 0.015). No significant differences were reported for days since last vaccination. Demographic characteristics and comorbidities are presented in Table 1.

**Table 1:** Participant characteristics.

Characteristics (n = 127)	Long COVID (n = 63)	Non-infected controls (n = 21)	Acutely infected controls (n = 22)	Spontaneous Resolvers (n = 21)
**Age (years), median (IQR)**	53 (14)	47 (33)	44 (29)	42 (44)*
**Sex n (%)**				
Male	18 (28%)	7 (33%)	10 (45%)	9 (43%)
Female	46 (72%)	14 (67%)	12 (55%)	12 (57%)
**Height (cm) (mean ± SD)**	166 ± 9	165 ± 10	171 ± 9	168 ± 10
**Weight (kg) (mean ± SD)**	79 ± 16	69 ± 14	76 ± 14	73 ± 15
**Body mass index (kg/m** ^**2**^ **) median (IQR)**	28 (8)	25 (5)	26 (4)	25 (5)
**Vaccinated n (%)**				
Yes	60 (95%)	21(100%)	21 (96%)	20 (95%)
No	0 (0%)	0 (0%)	1 (4%)	0 (0%)
Unknown	3 (5%)	N/A.	N/A.	1 (5%) ^†^
One dose	2 (3%)	0 (0%)	1 (4%)	0 (0%)
Two doses	3 (5%)	3 (14%)	3 (14%)	1 (5%)
Three doses	18 (30%)	9 (43%)	10 (46%)	17 (80%)
> Three doses	35 (58%)	9 (43%)	7 (32%)	2 (10%)
Unknown dose number	2 (3%)	N/A.	N/A.	N/A.
**Comorbidities n (%)**				
Yes	48 (76%)	10 (48%)	15 (68%)	10 (48%)
No	13 (21%)	11 (52%)	7 (32%)	10 (48%)
Unknown	2 (3%)	0 (0%)	0 (0%)	1 (4%)
Endocrine/diabetes	9 (14%)	1 (5%)	2 (9%)	2 (10%)
Renal	8 (13%)	0 (0%)	1 (5%)	0 (0%)
Cardiovascular	19 (30%)	5 (24%)	4 (18%)	1 (5%)
Gastrointestinal/Liver	17 (27%)	1 (5%)	5 (23%)	1 (5%)
Neurological/cerebrovascular	20 (32%)	1 (5%)	5 (23%)	1 (5%)
Malignancy including haematological	3 (5%)	3 (14%)	0 (0%)	0 (0%)
Respiratory	18 (29%)	1 (5%)	3 (14%)	4 (19%)
Rheumatological	7 (11%)	0 (0%)	1 (5%)	0 (0%)
Psychological	4 (6%)	0 (0%)	4 (18%)	0 (0%)
Other	13 (21%)	1 (5%)	5 (23%)	2 (10%)

*denotes data available for n = 20. † Participant was unable to determine the total number of vaccinations received or provide vaccination status within the timeframe of the study.

### Acute COVID-19

The number of COVID-19 infections was significantly higher in the acute group compared with the spontaneous resolvers group (2 [1] vs. 1 [1] infections; p = 0.008). There were no statistical differences reported between the Long COVID and spontaneous resolvers, or Long COVID and acute groups for number of infections. Days since last infection was significantly greater in the Long COVID (447 [502], range: 100–1347 days; p < 0.001) and spontaneous resolvers groups (451 [287], range: 133–833 days; p < 0.001) when compared with the acutely infected group (40 [27], range: 8–82 days). No statistical differences were observed between the Long COVID and spontaneous resolvers groups. Eight participants (13%) living with Long COVID were admitted to hospital during their acute infection; meanwhile, only one participant (2%) from the acutely infected group and zero participants from the spontaneous resolvers group were admitted to hospital due to their COVID-19 infection.

### Long COVID

Sixty-three participants meeting the WHO Long COVID definition were categorised into four groups based upon perceived severity using the PCFS. Accordingly, 9 (14%), 20 (32%), 24 (38%), and 10 (16%) participants were allocated to PCFS groups 4A, 4B, 4C, and 4D (4A – negligible limitations in everyday life, 4D – severe limitations in everyday life). The impact of symptoms on daily life was rated 3/10 (2), 5/10 (3), 8/10 (2), and 9/10 (2), for groups 4A-D respectively. Symptom prevalence across the Long COVID cohort was greatest for fatigue (92%), concentration issues (71%), and difficulty sleeping (70%) (Table 2). When classified into PCFS subgroups, fatigue remained the most prevalent symptom across all groups; however, in the most benign group (4A), headaches (67%) and chest tightness (56%) were the next most reported symptoms. Memory loss was also frequently reported from PCFS groups 4C (82%) and 4D (100%). Perceived severity using the PCFS scale aligned with symptom reporting as participants in group 4D reported symptoms more frequently when expressed as a percentage compared with group 4A (17/24 symptoms), 4B (22/24 symptoms), and 4C (14/24 symptoms). This trend also aligned with severity classification between groups 4A, 4B, and 4C (Table 2).

**Table 2:** Symptom reporting from people living with Long COVID sub-grouped using the PCFS.

	Long COVID				
Symptom	A (n = 9)	B (n = 19)	C (n = 22)	D (n = 9)	All (n = 59)
Cough	3 (33%)	4 (21%)	8 (36%)	2 (22%)	17 (29%)
Fatigue	6 (67%)	17 (90%)	22 (100%)	9 (100%)	54 (92%)
Chest pain	1 (11%)	3 (16%)	8 (36%)	3 (33%)	15 (25%)
Chest Tightness	5 (56%)	7 (37%)	11 (50%)	4 (44%)	27 (46%)
Memory loss	4 (44%)	9 (47%)	18 (82%)	9 (100%)	40 (68%)
Concentration	4 (44%)	11 (58%)	19 (86%)	8 (89%)	42 (71%)
Difficulty sleeping	4 (44%)	13 (68%)	17 (77%)	7 (78%)	41 (70%)
Dizziness	2 (22%)	6 (32%)	15 (68%)	6 (67%)	29 (49%)
Pins & needles	3 (33%)	8 (42%)	10 (46%)	6 (67%)	27 (46%)
Joint pain	2 (22%)	10 (53%)	13 (59%)	6 (67%)	31 (53%)
Depression/low mood	1 (11%)	3 (16%)	10 (46%)	5 (56%)	19 (32%)
Anxiety	2 (22%)	6 (32%)	13 (59%)	5 (56%)	26 (44%)
Tinnitus/earache	3 (33%)	8 (42%)	13 (59%)	4 (44%)	28 (48%)
Heart palpitations	3 (33%)	10 (53%)	13 (59%)	5 (56%)	31 (53%)
Nausea	2 (22%)	3 (16%)	7 (32%)	2 (22%)	14 (24%)
Diarrhoea	1 (11%)	2 (11%)	2 (9%)	1 (11%)	6 (10%)
Stomach ache	0 (0%)	3 (16%)	5 (23%)	3 (33%)	11 (19%)
Loss of appetite	0 (0%)	3 (16%)	4 (18%)	5 (56%)	12 (20%)
Temperature regulation	0 (0%)	3 (16%)	7 (32%)	4 (44%)	14 (24%)
Headache	6 (67%)	11 (58%)	17 (77%)	5 (56%)	39 (66%)
Sore throat	1 (11%)	4 (21%)	10 (46%)	3 (33%)	18 (31%)
Loss/change of smell	3 (33%)	3 (16%)	5 (23%)	2 (22%)	13 (22%)
Loss/change of taste	2 (22%)	3 (16%)	5 (23%)	2 (22%)	12 (20%)
Rashes	0 (0%)	0 (0%)	3 (14%)	3 (33%)	6 (10%)
Other	3 (33%)	12 (63%)	15 (68%)	7 (78%)	37 (63%)

### Biomarker findings

IL-12 (p40/p70) was significantly greater in the Long COVID (58 [49], min-max: 21–214 pg/mL; p = 0.046) and acutely infected (64 [36] min-max: 29–778 pg/mL; p = 0.039) groups when compared with non-infected controls (45 [25], min-max: 21–91 pg/mL) (Table 3 and Fig 1). Hepatocyte Growth Factor (HGF) was significantly greater in the spontaneous resolvers group when compared with non-infected controls (526 [345], min-max: 151–2111 pg/mL vs. 297 [229], min-max: 130–870 pg/mL; p = 0.027). No further statistically significant differences were observed between the groups for any of the cytokines examined (Table 3).

**Table 3:** Cytokine and chemokine data for Long COVID participant and three comparator groups.

	Non-infected controls			Acutely infected			Spontaneous Resolvers			Long COVID		
Cytokine	*n*	Median (IQR)	Range	*n*	Median (IQR)	Range	*n*	Median (IQR)	Range	*n*	Median (IQR)	Range
G-CSF	21	260 (482)	89-3612	22	227 (230)	70-3042	21	256 (763)	125-5916	63	192 (219)	83-3424
GM-CSF	21	15 (64)	2-592	18	81 (278)	1-1052	15	58 (162)	4-873	54	64 (208)	1-1728
IFN-α	17	31 (41)	1-195	14	27 (85)	4-299	20	12 (25)	1-179	41	16 (55)	1-523
IFN-γ	21	4 (2)	2-111	22	4 (1)	2-18	21	4 (1)	2-6	63	4 (1)	1-164
IL-1RA	20	255 (126)	65-442	21	193 (173)	54-3488	20	222 (176)	28-661	62	220 (197)	85-4087
IL-2	19	56 (67)	3-768	22	23 (38)	1-1673	20	19 (54)	1-625	61	10 (53)	1-2263
IL-2R	21	282 (1341)	68-11937	22	483 (950)	125-16002	21	350 (2263)	134-7843	63	317 (750)	98-16567
IL-4	21	16 (13)	9-37	22	16 (24)	5-1143	21	16 (17)	8-741	63	18 (16)	3-1527
IL-6	21	27 (98)	3-995	22	27 (45)	4-3028	21	18 (56)	5-714	63	14 (57)	4-2989
IL-7	13	16 (20)	2-52	13	13 (29)	5-265	13	8 (13)	1-195	43	14 (13)	4-128
IL-8	21	15 (10)	9-35	22	13 (8)	3-25	21	16 (11)	6-38	63	14 (13)	4-352
IL-10	21	10 (52)	3-281	22	18 (39)	2-2726	21	9 (29)	3-989	63	9 (49)	1-7162
IL-12 (p40/p70)	21	45 (25)	21-91	22	64 (36) ^*^	29-778	21	51 (32)	24-377	63	58 (49) ^*^	21-214
IL-13	21	17 (14)	2-129	22	17 (16)	8-2722	21	16 (15)	8-142	63	17 (24)	3-1931
IL-15	17	10 (22)	1-133	14	10 (109)	1-1403	18	6 (17)	0.2-1034	27	11 (77)	1-210
TNF-α	21	5 (46)	2-763	22	9 (19)	2-1321	21	10 (62)	2-484	63	6 (16)	2-1197
Eotaxin	21	63 (42)	33-105	22	64 (24)	32-140	21	72 (39)	11-589	63	71 (46)	22-164
IP-10 (CXCL10)	18	2 (2)	1-6	19	3 (2)	1-15	17	3 (4)	2-53	46	3 (3)	1-24
MCP-1	21	454 (221)	133-944	22	387 (228)	236-10037	21	434 (290)	132-4564	63	419 (201)	155-3681
MIG	16	12 (17)	3-76	15	9 (11)	2-51	19	10 (15)	1-868	41	9 (13)	1-668
MIP-1α	21	64 (89)	5-355	22	72 (59)	34-3648	21	87 (166)	25-2259	63	67 (68)	5-818
MIP-1β	21	387 (487)	63-3529	22	212 (223)	96-5605	21	221 (471)	49-2976	63	189 (352)	55-5815
RANTES (CCL5)	20	2372 (856)	1744-4986	22	2693 (1070)	2024-6736	20	2686 (768)	2002-3870	62	2611 (692)	1350-13651
EGF	21	136 (121)	48-964	22	122 (101)	46-1698	21	114 (158)	38-636	62	123 (85)	42-2210
FGF-basic	21	34 (27)	20-85	22	33 (14)	18-744	21	32 (24)	22-321	63	26 (11)	17-308
HGF	21	297 (229)	130-870	22	383 (220)	185-4105	21	526 (345) ^*^	151-2111	63	383 (244)	128-2209
VEGF-A	21	4 (8)	2-36	22	5 (5)	1-76	21	6 (9)	2-53	63	5 (5)	1-116

All cytokine units are pg/mL. ^*^Denotes p < 0.05 compared with non-infected controls.

**Fig 1:**
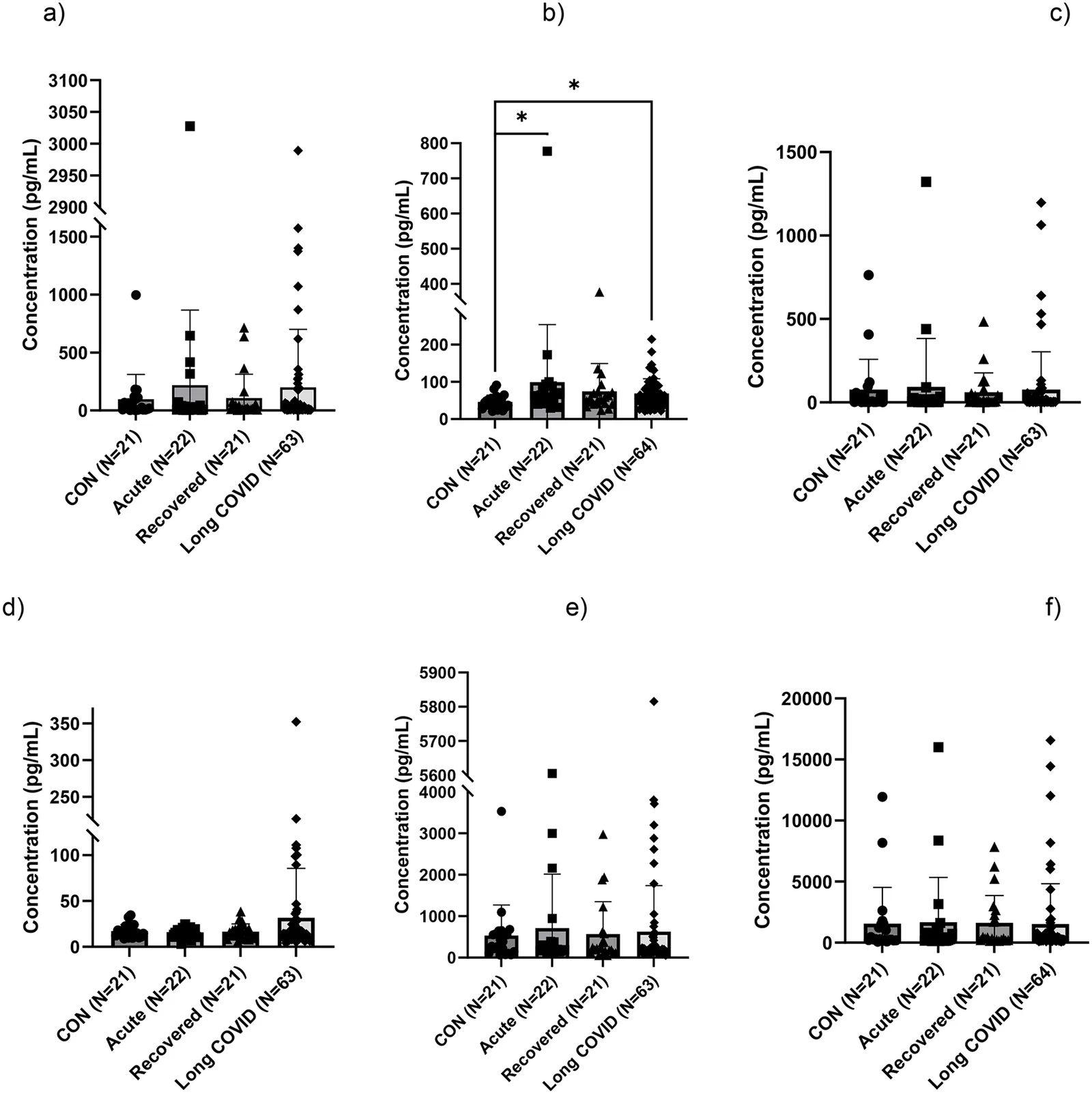
Cytokine data expressing the variable responses of groups for a) IL-6, b) IL-12 (p40/p70), c) TNF-α, d) IL-8, e) MIP-1β, f) IL-2R. *Denotes p < 0.05 compared with CON. CON denotes non-infected comparator group. Acute denotes acutely infected comparator group (<3 months). Recovered denotes recovered from SARS-CoV-2 comparator group (>3 months).

Following classification of the Long COVID cohort into PCFS subgroups, only basic fibroblast growth factor (FGF-basic) concentration was significantly lower in Long COVID group 4B when compared with the spontaneous resolvers group (24 [7], min-max: 17–123 pg/mL vs. 32 [24], min-max: 22–321 pg/mL; p = 0.025). Again, no further statistically significant differences were observed between the Long COVID subgroups and comparator groups for any of the cytokines examined (Table 4).

**Table 4:** Cytokine and chemokine data for Long COVID subgroups.

	PCFS A			PCFS B			PCFS C			PCFS D		
Cytokine	*n*	Median (IQR)	Range	*n*	Median (IQR)	Range	*n*	Median (IQR)	Range	*n*	Median (IQR)	Range
G-CSF	9	240 (969)	124-2536	20	176 (86)	83-3424	24	193 (211)	111-3175	10	178 (566)	119-2620
GM-CSF	9	60 (339)	6-1286	17	71 (109)	1-488	19	71 (333)	1-1728	9	46 (368)	1-1034
IFN-α	7	44 (68)	11-185	11	7 (21)	2-194	17	10 (51)	1-310	6	71 (262)	1-523
IFN-γ	9	4 (1)	3-5	20	3 (1)	2-8	24	4 (1)	1-8	10	5 (2)	3-164
IL-1β	8	3 (10)	2-745	13	3 (1)	2-315	16	4 (4)	1-37	10	3 (8)	2-63
IL-1RA	9	238 (414)	136-3661	20	194 (137)	107-1836	23	203 (152)	95-558	10	390 (768)	85-4087
IL-2	9	21 (325)	5-771	18	8 (28)	2-932	24	11 (28)	1-1036	10	11 (655)	6-2263
IL-2R	9	351 (3899)	141-14439	20	308 (479)	98-16567	24	319 (710)	118-12024	10	288 (2307)	117-8182
IL-4	9	7 (13)	4-1521	20	18 (10)	3-1527	24	19 (11)	6-86	10	14 (164)	3-1039
IL-6	9	16 (323)	6-870	20	11 (37)	5-1571	24	15 (156)	5-2989	10	14 (351)	4-1070
IL-7	4	36 (49)	11-63	14	10 (7)	6-58	19	15 (12)	4-77	6	23 (80)	13-128
IL-8	9	14 (9)	6-19	20	14 (14)	4-352	24	17 (26)	8-220	10	12 (5)	10-100
IL-10	9	8 (20)	4-1240	20	6 (29)	2-185	24	21 (169)	1-7162	10	30 (150)	2-1302
IL-12 (p40/p70)	9	63 (62)	26-180	20	54 (55)	22-146	24	57 (36)	32-214	10	74 (49)	21-136
IL-13	9	16 (42)	5-1430	20	15 (15)	3-1931	24	19 (27)	8-190	10	23 (27)	10-541
IL-15	2	84 (5)	82-87	7	9 (20)	1-153	14	8 (94)	1-210	4	19 (64)	3-80
IL-17A	7	8 (24)	3-398	4	4 (397)	1-528	9	14 (63)	2-114	4	4 (52)	1-69
TNF-α	9	6 (274)	2-640	20	5 (9)	2-1197	24	6 (16)	2-1064	10	4 (17)	2-531
Eotaxin	9	62 (33)	33-77	20	74 (48)	22-131	24	74 (54)	27-164	10	49 (49)	35-147
IP-10 (CXCL10)	8	3 (2)	1-24	11	2 (4)	1-7	18	3 (3)	1-9	9	4 (4)	1-9
MCP-1	9	419 (256)	178-594	20	397 (224)	224-646	24	458 (326)	155-879	10	404 (158)	184-3681
MIG	6	5 (14)	1-37	10	7 (11)	1-29	18	11 (18)	2-146	7	12 (34)	3-668
MIP-1α	9	44 (63)	20-167	20	54 (55)	5-200	24	81 (64)	35-818	10	61 (112)	38-238
MIP-1β	9	269 (1456)	115-3197	20	158 (145)	55-3710	24	216 (318)	95-3804	10	144 (2296)	85-5815
RANTES (CCL5)	9	3016 (1266)	1995-13651	19	2468 (554)	1350-3180	24	2611 (755)	1417-5750	10	2704 (509)	2246-8269
EGF	9	155 (408)	43-1039	19	105 (71)	42-1397	24	126 (82)	65-1065	10	114 (604)	60-2210
FGF-basic	9	32 (23)	26-107	20	24 (7)^*^	17-123	24	27 (18)	18-144	10	29 (127)	23-308
HGF	9	345 (138)	228-509	20	323 (284)	128-765	24	395 (303)	136-1322	10	429 (367)	157-2209
VEGF-A	9	6 (31)	2-75	20	5 (5)	1-116	24	6 (8)	2-102	10	6 (8)	1-43

All cytokine units are pg/mL. PCFS; post-COVID-19 functional status scale. ^*^Denotes p < 0.05 compared with recovered compactor group.

### Correlational analysis

Group-specific correlation matrices revealed strong positive associations (r > 0.5) between cytokines for all groups (Fig 2). Very strong positive associations (r > 0.8) between cytokines were reported for eleven (5%), eight (4%), twelve (6%), seventeen (8%), six (3%), eight (4%), and twelve (6%) pairings in the non-infected, acutely infected, spontaneous resolvers, and Long COVID groups A-D, respectively (excluding same cytokine pairing). Of these pairings, TNF-α-IL-2R strongly associated across all groups. Long COVID subgroups displayed heterogeneous correlation patterns with strong positive associations.

**Fig 2:**
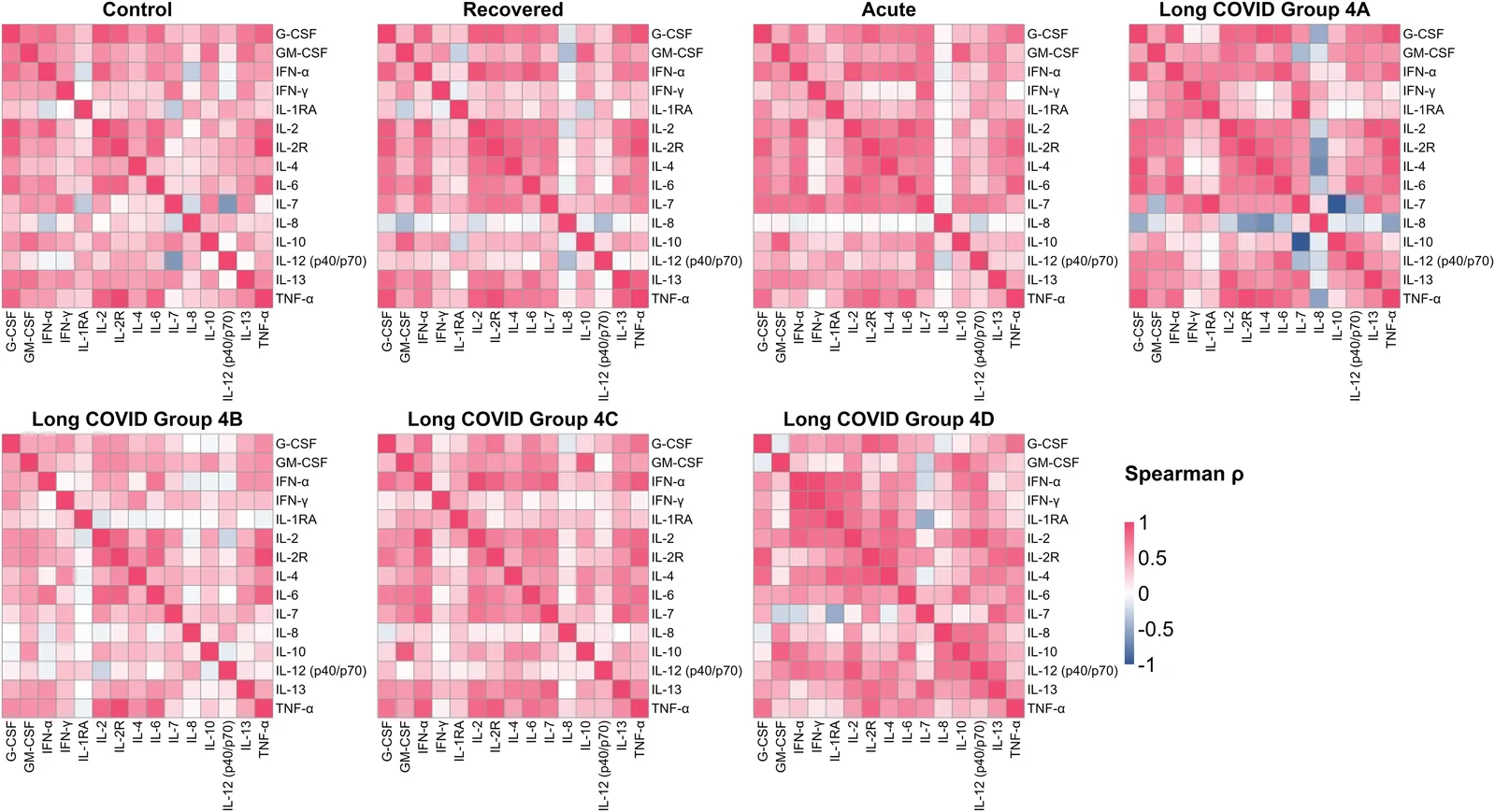
Spearman rank correlations (ρ) visualised through correlation matrices using fixed-scale heatmaps (−1 to 1) with identical cytokine ordering across groups. Control denotes non-infected comparator group. Acute denotes acutely infected comparator group (<3 months). Recovered denotes spontaneous resolvers that have recovered from SARS-CoV-2 comparator group (>3 months).

Fig 3 illustrates the pairwise correlation differences between the non-infected control group with all other remaining groups. Here, Long COVID groups C & D demonstrate greater positive cytokine associations compared with the non-infected control group suggesting a coordinated inflammatory response. Similar but more subdued differences were identified in Long COVID group A and the acutely infected group, with a greater frequency of more negative cytokine associations observed in Long COVID group A compared to the non-infected control group. Very few positive differences were observed for Long COVID group 4B as opposed to the other three Long COVID subgroups when compared to non-infected controls. Long COVID group B demonstrated only one strong positive cytokine pairing difference (IL-7-IL-12(p40/70) with several more negative differences reported for other cytokine associations.

**Fig 3:**
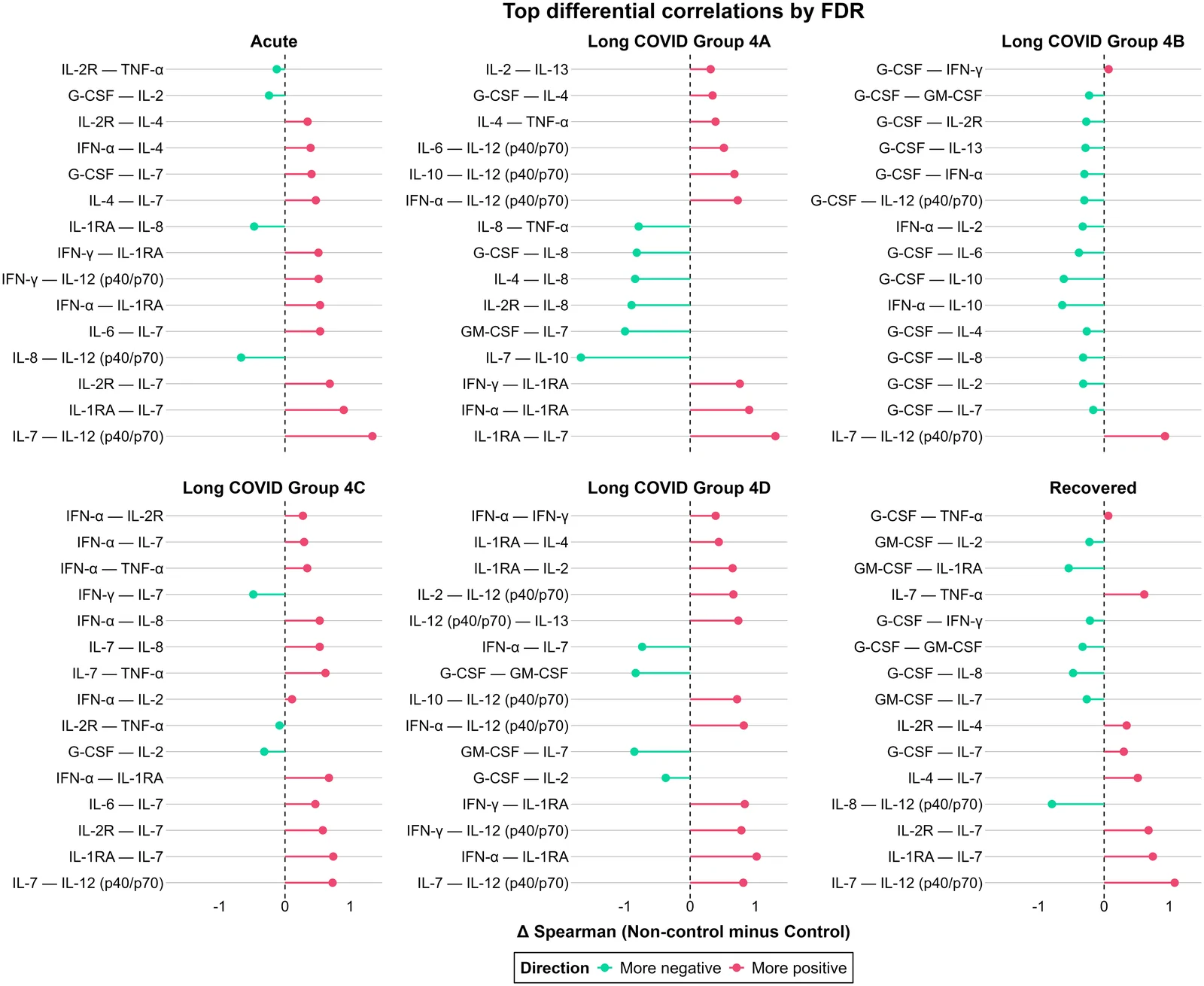
Differences in pairwise cytokine correlations between the non-infected control group with all other remaining groups with Benjamini–Hochberg false discovery rate (FDR) adjustments made for multiple comparisons. Acute denotes acutely infected comparator group (<3 months). Recovered denotes spontaneous resolvers that have recovered from SARS-CoV-2 comparator group (>3 months).

### Principal component analysis (PCA)

The first principal component accounted for the largest proportion of variance in the cytokine dataset; however, PCA did not reveal discernible clustering by clinical group (Fig 4). Statistical comparisons of PC1 scores between non-infected control and non-control groups were not significant following multiple testing correction. Overall, these results indicate that dominant axes of coordinated cytokine variation are not clearly group specific.

**Fig 4:**
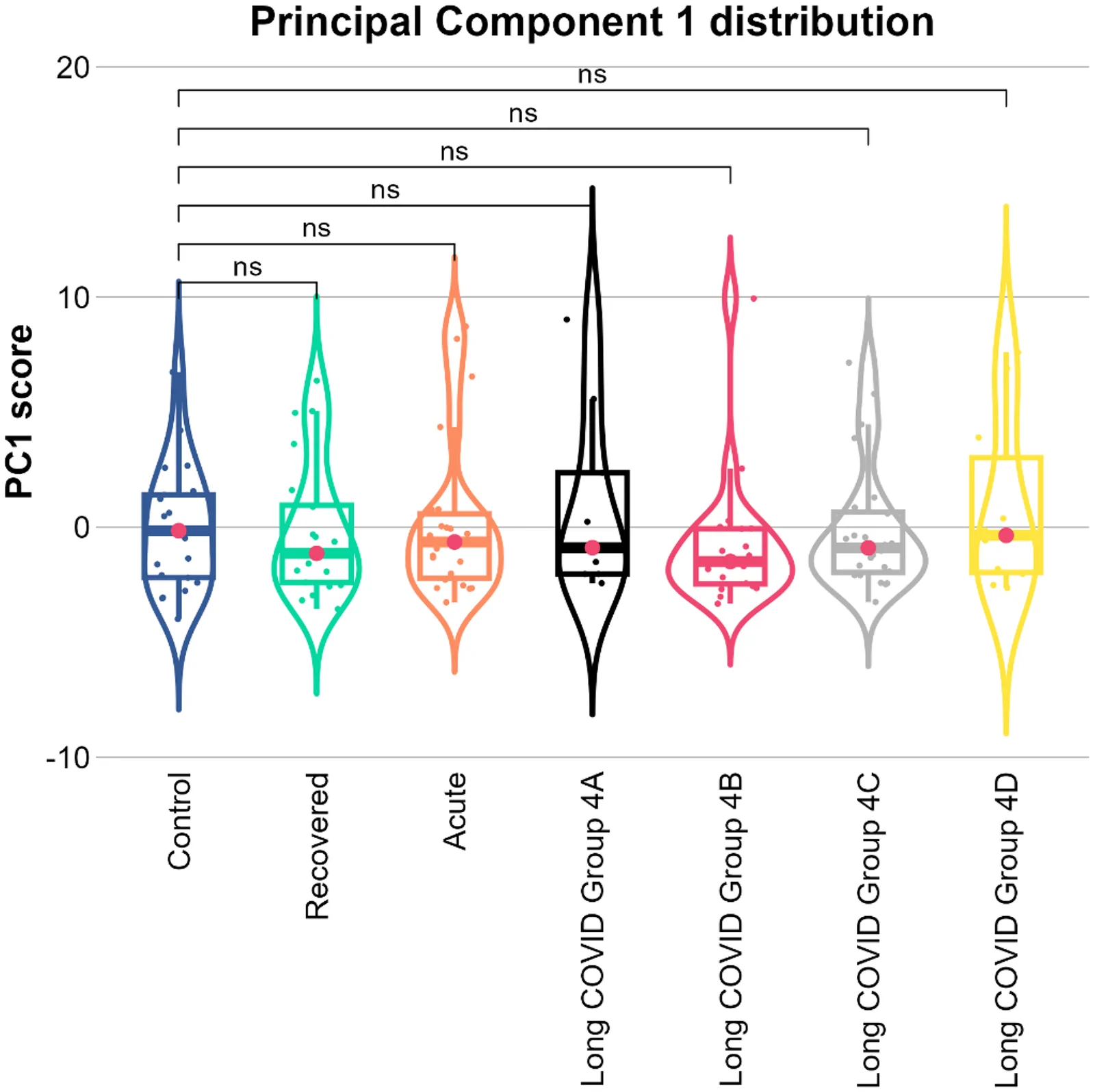
Principal component 1 (PC1) scores that compare the control group and each non-control group using two-sided Wilcoxon rank-sum tests, with p-values adjusted using the Benjamini–Hochberg procedure. Control denotes non-infected comparator group. Acute denotes acutely infected comparator group (<3 months). Recovered denotes spontaneous resolvers that have recovered from SARS-CoV-2 comparator group (>3 months).

## Discussion

This study aimed to understand the role of candidate Long COVID blood biomarkers and determine whether PCFS severity stratification was reflected through these measures. This study found that comparing blood biomarkers that have roles within immunological and inflammatory function, and vascular growth and repair do not reveal any clear diagnostic Long COVID biomarkers when compared to varied comparator groups at a single timepoint. These findings extend to when the PCFS is used to classify severity in those living with Long COVID, providing evidence against the use of perceptual classification tools or symptom frequency to quantify biological severity. Pairwise correlation differences better reflected Long COVID status and severity compared to non-infected controls (e.g., coordinated inflammatory response); however, PCA was unable to determine any coordinated cytokine variation that was clearly group specific. Significant biomarker concentration ranges were observed in this study, and whilst these biomarkers may not serve as diagnostic targets, they could help guide clinical decision making with regards to rehabilitative and therapeutic approaches. For example, those with significantly greater inflammatory biomarker profiles may warrant anti-inflammatory therapies or further investigations to identify the cause [14,15].

During the acute phase of severe COVID-19 infections, several pro- and anti-inflammatory markers contribute to the commonly labelled cytokine storm (e.g., Monocyte Chemoattractant Protein-1 [MCP-1], epidermal growth factor [EGF], Interferon-gamma inducible protein 10 [IP-10], IL-8, TNF-α, Eotaxin, and FGF-basic) [16–19]. The cytokine storm has significant implications not only for acute phase complications, but heightened and prolonged inflammation and immunological activation which are central to the proposed mechanisms of Long COVID. For example, acute phase biomarkers FGF-basic, EGF, IL-12(p70), IL-6, IL-13, VEGF-A have all been associated with the development of Long COVID [16]. IL-12 p40 is produced by immune cells (e.g., macrophages and dendritic cells) and is a natural antagonist with IL-12 p70 (considered the biologically active form of IL-12) that competes to bind with the IL-12 receptor [20]. In the present study, IL-12 (p40/p70) was the only biomarker that was significantly greater in the Long COVID cohort versus a comparator group (non-infected controls), and this may be indicative that those living with Long COVID are trying to regulate an ongoing inflammatory response. Pairwise correlation differences from three of the Long COVID subgroups support this notion; however, there was a paucity of other statistically elevated inflammatory blood biomarkers in the Long COVID cohort when compared to the non-infected controls and spontaneous resolvers in this study. Fig 1 reveals the range of several key inflammatory cytokines and chemokines between groups, and it is apparent that there are those in the Long COVID cohort that have substantially greater concentrations. Furthermore, those with greater concentrations were often different individuals living with Long COVID whereas one outlier from the non-infected control group, and two from the spontaneous resolvers group, were data from the same three participants. The non-infected individual with elevated markers had no stated comorbidities but did report taking hormonal birth control, which was not received by any other control participant. This medication may explain the increased inflammation as some associations have been made between hormonal contraception and inflammatory markers (e.g., TNF-α) [21]. The spontaneous resolvers group included two young (29 years old) females with no comorbidities or active treatments/medications reported but it may be possible that the participants did not disclose information regarding hormonal contraception as all demographic information were self-report. Another plausible explanation is that the menstrual cycle phase may have influenced these markers. Generally, it is assumed that the follicular phase and oestrogen seem to exert more anti-inflammatory effects; meanwhile, the luteal phase seemingly exert more pro-inflammatory effects which is where these individuals could have been in their cycle [22,23]. These findings reaffirm the benefits of detailed comparator groups, which could be improved further with consented access to health records. The broad number of responders in the Long COVID group for heightened inflammation could help inform therapeutic and treatment trials, offering a potential benefit to those whose symptoms may be driven by this factor.

One common theme observed from Long COVID research is the lack of consistent and validated blood biomarkers, and this seemingly extends to when participants are grouped by perceived severity. FGF-basic has a role in tissue repair and angiogenesis and increased concentrations have been observed in those living with Long COVID out to 7-months post-SARS-CoV-2 infection [24]. Phillipe *et al.* [25] also observed greater concentrations of FGF-basic in those who went on to develop Long COVID when compared to healthy volunteers during acute infection but showed how this biomarker progressively decreased from 3- to 24-months post-infection in the Long COVID group. Similarly, increased angiogenetic blood biomarkers (e.g., Angiopoietin-1 and P-selectin) have been detected in those living with Long COVID with laudable accuracy and reproducibility [26–28]. In the present study, FGF-basic was the only biomarker that was significantly different with the addition of the Long COVID PCFS subgroup analysis; however, FGF-basic was observed in smaller concentrations in Long COVID group B when compared with the spontaneous resolvers group. Pairwise correlation differences also identified subgroup group 4B as an outlier whereby the other three subgroups demonstrated strong, positive associations between cytokines when compared to non-infected controls, unlike Long COVID subgroup 4B. Whilst this analysis may indicate a form of coordinated inflammatory-type subgroup, it was not supported by the findings of the PCA. One alternative thought process as to the lack of distinctive biomarkers between Long COVID subgroups, and Long COVID and comparator groups, is that biomarkers may be reflective of the undulating and heteroscedastic symptom profiles people living with Long COVID exhibit. Specifically, Owen *et al.* [7] observed this phenomenon through questionnaire scales and functional assessments across a 16-week period with regular contact (every 2 weeks). To date, most blood biomarker studies have examined participants at either a single timepoint or, if repeated measures were taken, these were separated by large intervals that may possibly miss blood biomarker fluctuations. Future studies should consider a longitudinal approach with regular sample draws assessing commonly dysregulated blood biomarkers identified from previous reviews [2–4] alongside objective physiological measures which can be captured through wearable devices (e.g., heart rate variability). This may possibly identify symptom exacerbation and post-exertional malaise episodes that could then be coupled with blood biomarker fluctuations to better understand these pathophysiological mechanisms and Long COVID grouping.

An unexpected finding from this study was the lack of significant blood biomarkers that could distinguish those living with Long COVID when compared to the three different comparator groups. Whilst two biomarkers were identified, the rationale for panel selection based upon previous reviews of Long COVID blood biomarkers make the findings seemingly unexpected alongside the data from the acutely infected groups with considerable research demonstrating substantial increase in several inflammatory cytokines during acute SARS-CoV-2 infection [18,19,29]. Median time since last infection was beyond one month in the acute group, and the severity of acute infection across these participants was seemingly mild with only one participant admitted to hospital, which may explain the subdued response in this group. This study corroborates existing literature whereby there is an absence of a universally accepted diagnostic Long COVID blood biomarker [30]. The reliance on self-reported data, along with the heterogeneous and fluctuating nature of symptoms and functional status (e.g., the variability in participant condition at the time of recruitment), introduced significant methodological challenges. These factors can substantially limit the statistical power necessary for robust subgroup analyses, particularly in studies with modest sample sizes. Consequently, the adoption of alternative methodologies that consistently and accurately reflect the underlying pathophysiology of Long COVID may offer greater potential for advancing diagnostic precision.

### Limitations

The present study assessed blood biomarkers from participants at a single-timepoint providing only a snapshot of inflammatory, immunological, and vascular function on that day. It may be possible that longitudinal testing, possibly with the introduction of a stimulus to assess normal physiological function, would better help understand the individual and their ‘normal ranges’ as well as providing some diagnostic and mechanistic information. Variation in comorbidity profile between Long COVID and comparator groups, with a greater proportion reported in the former, may have influenced some inflammatory biomarkers. However, these comorbidities may have predisposed these individuals or manifested after SARS-CoV-2 infection and form part of the Long COVID sequalae. Future studies may consider comparator groups with known isolated comorbidities in attempt to better define Long COVID biomarkers from other conditions. Greater recruitment to improve the statistical power of subclassification analysis of the PCFS could help improve future studies. Target recruitment was initially much higher for the most benign and severe Long COVID participants in this study (n = 20); however, many of those living with the most severe limitations were unable to attend the data collection site. Furthermore, it may be possible those living with the most benign form of Long COVID were insufficiently motivated or interested in taking part in this work if symptoms only negligibly impacted activities of daily living. This study did not record exact data as to whether those living with Long COVID or comparator groups who had recently been infected with, or had recovered from, SARS-CoV-2 were confirmed via PCR test, lateral flow test or retrospective clinical confirmation. Uncertainty also surrounded whether the non-infected participants had never unknowingly contracted COVID-19, and some studies may consider PCR testing to verify controls or use blood biobank samples taken prior to 2019. However, PCR testing does not guarantee the absence of past COVID-19 infection due to antibody waning [31] and suitable biobank samples with sufficient participant information may not be readily available or affordable. This study included cytokine values that were below the LLOQ for the assay to preserve biological validity of the dataset; however, these values are influenced by extrapolation and could make the data unreliable. Future approaches may consider imputation (e.g., replacing LLOQ data with LLOQ/2 values), sensitivity analysis with larger datasets to assess the impact of performing the analysis with and without the LLOQ values, or an assay redesign by including high-sensitivity ELISAs to determine key cytokine concentrations where LLOQ values are frequently reported. The latter may also improve the sample size of key cytokine analysis as out-of-range values decreased the group size comparisons for individual cytokines within the present study which accounts for the inconsistent participants numbers in Tables 3 and 4.

## Conclusions

This study found that only IL-12 (p40/p70) and FGF-basic, which reflect ongoing inflammation and tissue repair and/or angiogenesis respectively, were significantly elevated in either the Long COVID group or subgroup against non-Long COVID comparators at single timepoint. This work reaffirms the lack of diagnostic Long COVID biomarkers and limitations of taking a broad approach that does not take into consideration the inter-individual variability and episodic symptom nature of those living with Long COVID. Future research should aim to capture these traits within blood biomarker profiles and make use of wearable technologies to help provide context of the individual across longitudinal and regular timepoints.

## Supporting information

S1 Table The post-COVID-19 Functional Status Scale describes and categorises those recovering from a SARS-CoV-2 infection based upon functional limitations. (DOCX)

S1 File Supporting information with the dataset of the present study. (XLSX)
