# Supplementary material for: Blood biomarkers of long COVID: A cross-sectional evaluation of key cytokines and the coordination of inflammation
Source: PLoS One. 2026 Oct 21;21(10):e0360525. doi: 10.1371/journal.pone.0360525 (PMC13686509; doi:10.1371/journal.pone.0360525)
Supplement: Supplementary file 1 [file pone.0360525.s001.docx]

**S1.** **The post-COVID-19 Functional Status Scale describes and categorises those recovering from a SARS-CoV-2 infection based upon functional limitations.**

| How much are you currently affected in your everyday life by COVID-19?  Please indicate which one of the following statements applies to you most. *Please tick only one box at a time* |  | Corresponding PCFS scale grade  if the box is ticked |
| --- | --- | --- |
| I have negligible limitations in my everyday life as I can perform all usual duties/activities, although I still have persistent symptoms, pain, depression, or anxiety. | □ | 1 |
| I suffer from limitations in my everyday life as I occasionally need to avoid or reduce usual duties/activities or need to spread these over time due to symptoms, pain, depression or anxiety. I am, however, able to perform all activities without any assistance. | □ | 2 |
| I suffer from limitations in my everyday life as I am not able to perform all usual duties/activities due to symptoms, pain, depression, or anxiety. I am, however, able to take care of myself without any assistance. | □ | 3 |
| I suffer from severe limitations in my everyday life: I am not able to take care of myself and therefore I am dependent on nursing care and/or assistance from another person due to symptoms, pain, depression, or anxiety. | □ | 4 |
