# Supplementary material for: Blood biomarkers of long COVID: A cross-sectional evaluation of key cytokines and the coordination of inflammation
Source: PLoS One. 2026 Oct 21;21(10):e0360525. doi: 10.1371/journal.pone.0360525 (PMC13686509; doi:10.1371/journal.pone.0360525)
Supplement: Supplementary file 2 [file pone.0360525.s004.docx]

**Response to Editor and Reviewers**

"Blood Biomarkers of Long COVID: A Cross-Sectional Evaluation of Key Cytokines and the Coordination of Inflammation." (PONE-D-26-23542).

Dear Editor and Reviewers,

Thank you for your comments. My co-authors and I have revised the manuscript according to the feedback provided, which we think has improved the manuscript. For convenience, we have included below a table with point-by-point responses to editor and reviewers’ comments.

If there are any further requirements from our research team, then please do not hesitate to ask, and we sincerely thank you for considering our revised manuscript.

Kind regards,

Callum Thomas.

| **Comment addressed** | **Response** | **Location of amendment** | |
| --- | --- | --- | --- |
| Editor responses | | | |
| Please ensure that your manuscript meets PLOS ONE's style requirements, including those for file naming. | We have amended our manuscript so that it now aligns with the journal requirements sent via the links. Thank you providing those specific requirements. | | Throughout manuscript. |
| In the online submission form you indicate that your data is not available for proprietary reasons and have provided a contact point for accessing this data. Please note that your current contact point is a co-author on this manuscript. According to our Data Policy, the contact point must not be an author on the manuscript and must be an institutional contact, ideally not an individual. Please revise your data statement to a non-author institutional point of contact, such as a data access or ethics committee, and send this to us via return email. Please also include contact information for the third party organization, and please include the full citation of where the data can be found. | A minimal dataset has now been uploaded as a supporting information file (S2) as per the Journal Policy. We have also amended and identified this in the data availability statement. | | S2 |
| Your ethics statement should only appear in the Methods section of your manuscript. If your ethics statement is written in any section besides the Methods, please delete it from any other section. | The ethics statement that originally followed the acknowledgement has now been removed, with the ethics statement now only appearing in the methods. | | Page 5 Line 110 and Page 22, Line 467. |
| We notice that your supplementary tables are included in the manuscript file. Please remove them and upload them with the file type 'Supporting Information'. Please ensure that each Supporting Information file has a legend listed in the manuscript after the references list. | Supporting Informaiton Table 1 (S1) has now been removed at the end of the manuscript file and uploaded as *S1*. A legend for S1 has been included after the references list. | | Page 24, Lines 557-561 and S1. |
| Reviewer #1 responses | | | |
| Many biomarkers and group comparisons were tested, but only a small number of significant findings were reported. Some p-values are close to 0.05, so these results may be due to chance. The authors should explain more clearly how multiple comparisons were controlled for in all analyses, not only in the correlation analysis. | Multiple comparisons were controlled for using Dunn’s corrections and is stated in the statistical analyses we conducted for the Kruskal-Wallis tests completed on all data as this was all nonparametric. We have included some minor amendments into this section to make this clearer to the reader. | Page 6, Lines 166-167. | |
| The conclusions should be more cautious. The data support the finding that no clear diagnostic cytokine profile was identified. However, the claims about persistent immune activation and biological Long COVID subgroups are not fully proven by this study. These findings should be described as exploratory and should be confirmed in larger, longitudinal studies. | We thank the reviewer for their comments and recognise the size of p values falling just under the 0.05 alpha level for their previous comment and have now amended the *Discussion and Conclusions* section of the abstract to reflect these findings and the reviewers’ suggestions. | Page 2, Lines 49-53. | |
| The authors should also provide the minimal dataset in a public repository, or clearly explain why public sharing is not possible | A minimal dataset has now been uploaded as a supporting information file (S2) as per the Journal Policy. We have also amended and identified this in the data availability statement. | S2. | |
| Reviewer #2 responses | | | |
| Explain how the sample size for each group was calculated, particularly given the small number of participants in the control and PCFS subgroups, and whether an a priori power calculation was performed. | Given the exploratory nature of the analysis, we had not conducted a formal sample size calculation for the included groups; however, a sample size target of twenty participants for each group and each PCFS subgroup was considered appropriate given the available literature at the time of study design which were including a minimum of ten participants for exploratory studies. We have now amended our methods to state that no formal sample size calculation was completed and included the above information for context. | Page 5, Line 112 to Line 115. | |
| The Long COVID and control groups appear to differ in their comorbidity profiles, which may affect inflammatory biomarkers. Please explain why comorbidities were more common in the Long COVID group and whether this difference could have influenced the biomarker results. | We thank the reviewer for their comments and recognise the difference in comorbidity profile presented in Table 1. A factor that may contribute towards this is included in our methods where, as part of our exclusion criteria, we aimed to mitigate the issue of contributing comorbidities of comparator groups by excluding comparator participants with any known comorbidities or health conditions (e.g. inflammatory arthritis, autoimmune conditions, chronic obstructive pulmonary disease, etc.) that would interfere with sample analysis with the exception of Long COVID participants as we believed these conditions may have contributed to or manifested following their SARS-CoV-2 infection and form part of the Long COVID condition.  We have now recognised this within our limitations section and suggested a way forward for future work. | Page 20, Lines 428-433. | |
| Describe how SARS-CoV-2 infection was confirmed in each group, including how many participants were confirmed by PCR, lateral flow testing, or retrospective clinical confirmation. | We unfortunately do not have specific data as to whether participants were confirmed by PCR, lateral flow testing, or retrospective clinical confirmation. However, we have amended our manuscript to state both comparator groups 2 and 3 had a confirmed test via either PCR or lateral flow.  We have also amended our limitations section to recognise we do not have data to distinguish participants based upon confirmation route. | Page 5, Lines 125-128.  Page 21, Lines 439-441. | |
| There are inconsistencies in the participant numbers reported in Table 3; please explain the reasons for these discrepancies and the actual denominator used for each analysis. | Table 3 often reports different participant numbers for individual cytokines as these reflect results which were out of range and these data were not available for the statistical analysis. We have additionally recognised this within our limitations. | Page 21, Lines 452-454. | |
