# Supplementary material for: Blood biomarkers of long COVID: A cross-sectional evaluation of key cytokines and the coordination of inflammation
Source: PLoS One. 2026 Oct 21;21(10):e0360525. doi: 10.1371/journal.pone.0360525 (PMC13686509; doi:10.1371/journal.pone.0360525)
Supplement: Peer review history

# Peer review: Decision Letter 0

**DOI:** 10.1371/journal.pone.0360525.r001

**Authors:** Kota V Ramana

1 Sep 2026

Dear Dr. Faghy,

Thank you for submitting your manuscript to PLOS One. After careful consideration, we feel that it has merit but does not fully meet PLOS One’s publication criteria as it currently stands. Therefore, we invite you to submit a revised version of the manuscript that addresses the points raised during the review process.

Please submit your revised manuscript by Oct 31 2026 11:59PM. If you will need more time than this to complete your revisions, please reply to this message or contact the journal office at plosone@plos.org. When you're ready to submit your revision, log on to https://www.editorialmanager.com/pone/ and select the 'Submissions Needing Revision' folder to locate your manuscript file.

- A letter that responds to each point raised by the academic editor and reviewer(s). You should upload this letter as a separate file labeled 'Response to Reviewers'.
- A marked-up copy of your manuscript that highlights changes made to the original version. You should upload this as a separate file labeled 'Revised Manuscript with Track Changes'.
- An unmarked version of your revised paper without tracked changes. You should upload this as a separate file labeled 'Manuscript'.

If you would like to make changes to your financial disclosure, please include your updated statement in your cover letter. Guidelines for resubmitting your figure files are available below the reviewer comments at the end of this letter.

If applicable, we recommend that you deposit your laboratory protocols in protocols.io to enhance the reproducibility of your results. Protocols.io assigns your protocol its own identifier (DOI) so that it can be cited independently in the future. For instructions see: https://journals.plos.org/plosone/s/submission-guidelines#loc-laboratory-protocols. Additionally, PLOS One offers an option for publishing peer-reviewed Lab Protocol articles, which describe protocols hosted on protocols.io. Read more information on sharing protocols at https://plos.org/protocols?utm_medium=editorial-email&utm_source=authorletters&utm_campaign=protocols.

As the corresponding author, your ORCID iD is verified in the submission system and will appear in the published article. PLOS supports the use of ORCID, and we encourage all coauthors to register for an ORCID iD and use it as well. Please encourage your coauthors to verify their ORCID iD within the submission system before final acceptance, as unverified ORCID iDs will not appear in the published article. *Only*  the individual author can complete the verification step; PLOS staff *cannot*  verify ORCID iDs on behalf of authors.

We look forward to receiving your revised manuscript.

Kind regards,

Kota V Ramana, Ph.D.

Academic Editor

PLOS One

Journal Requirements:

When submitting your revision, we need you to address these additional requirements.

1. Please ensure that your manuscript meets PLOS ONE's style requirements, including those for file naming. The PLOS ONE style templates can be found at

https://journals.plos.org/plosone/s/file?id=wjVg/PLOSOne_formatting_sample_main_body.pdf and

https://journals.plos.org/plosone/s/file?id=ba62/PLOSOne_formatting_sample_title_authors_affiliations.pdf.

2. In the online submission form you indicate that your data is not available for proprietary reasons and have provided a contact point for accessing this data. Please note that your current contact point is a co-author on this manuscript. According to our Data Policy, the contact point must not be an author on the manuscript and must be an institutional contact, ideally not an individual. Please revise your data statement to a non-author institutional point of contact, such as a data access or ethics committee, and send this to us via return email. Please also include contact information for the third party organization, and please include the full citation of where the data can be found.

3. Your ethics statement should only appear in the Methods section of your manuscript. If your ethics statement is written in any section besides the Methods, please delete it from any other section.

4. We notice that your supplementary tables are included in the manuscript file. Please remove them and upload them with the file type 'Supporting Information'. Please ensure that each Supporting Information file has a legend listed in the manuscript after the references list.

5. If the reviewer comments include a recommendation to cite specific previously published works, please review and evaluate these publications to determine whether they are relevant and should be cited. There is no requirement to cite these works unless the editor has indicated otherwise.

[Note: HTML markup is below. Please do not edit.]

Reviewers' comments:

Reviewer's Responses to Questions

**Comments to the Author**

1. Is the manuscript technically sound, and do the data support the conclusions?

Reviewer #1: Partly

Reviewer #2: Yes

**********

2. Has the statistical analysis been performed appropriately and rigorously?

Reviewer #1: No

Reviewer #2: Yes

**********

3. Have the authors made all data underlying the findings in their manuscript fully available?

The PLOS Data policy

Reviewer #1: No

Reviewer #2: Yes

**********

4. Is the manuscript presented in an intelligible fashion and written in standard English?

Reviewer #1: Yes

Reviewer #2: Yes

**********

Reviewer #1: This is an interesting and relevant study. The manuscript is generally clear, and the authors included several comparison groups. The laboratory methods are also described well.

There are important limitations. The study is cross-sectional, so it cannot show whether the biomarker changes are caused by Long COVID or whether they change over time. Some Long COVID subgroups are very small, which reduces the reliability of the comparisons. The groups also differ in age, comorbidities, previous hospitalization, and time since infection. These factors may affect cytokine levels, but they were not fully adjusted for in the analysis.

Many biomarkers and group comparisons were tested, but only a small number of significant findings were reported. Some p-values are close to 0.05, so these results may be due to chance. The authors should explain more clearly how multiple comparisons were controlled for in all analyses, not only in the correlation analysis.

The conclusions should be more cautious. The data support the finding that no clear diagnostic cytokine profile was identified. However, the claims about persistent immune activation and biological Long COVID subgroups are not fully proven by this study. These findings should be described as exploratory and should be confirmed in larger, longitudinal studies.

The authors should also provide the minimal dataset in a public repository, or clearly explain why public sharing is not possible

Reviewer #2: 1. Explain how the sample size for each group was calculated, particularly given the small number of participants in the control and PCFS subgroups, and whether an a priori power calculation was performed.

2. The Long COVID and control groups appear to differ in their comorbidity profiles, which may affect inflammatory biomarkers. Please explain why comorbidities were more common in the Long COVID group and whether this difference could have influenced the biomarker results.

3. Describe how SARS-CoV-2 infection was confirmed in each group, including how many participants were confirmed by PCR, lateral flow testing, or retrospective clinical confirmation.

4. There are inconsistencies in the participant numbers reported in Table 3; please explain the reasons for these discrepancies and the actual denominator used for each analysis.

**********

what does this mean?). If published, this will include your full peer review and any attached files.

If you choose “no”, your identity will remain anonymous but your review may still be made public.

**Do you want your identity to be public for this peer review?**  For information about this choice, including consent withdrawal, please see our Privacy Policy

Reviewer #1: **Yes:**  Yuriy Bisyuk

Reviewer #2: No

**********

[NOTE: If reviewer comments were submitted as an attachment file, they will be attached to this email and accessible via the submission site. Please log into your account, locate the manuscript record, and check for the action link "View Attachments". If this link does not appear, there are no attachment files.]

To ensure your figures meet our technical requirements, please review our figure guidelines: https://journals.plos.org/plosone/s/figures

You may also use PLOS’s free figure tool, NAAS, to help you prepare publication quality figures: https://journals.plos.org/plosone/s/figures#loc-tools-for-figure-preparation.

NAAS will assess whether your figures meet our technical requirements by comparing each figure against our figure specifications.

# Author comment: Author response to Decision Letter 1

**DOI:** 10.1371/journal.pone.0360525.r002

9 Sep 2026

Dear Editor and Reviewers,

Thank you for your comments. My co-authors and I have revised the manuscript according to the feedback provided, which we think has improved the manuscript. For convenience, we have included below a table with point-by-point responses to the editor's and reviewers’ comments as an attachment to the submission.

If there are any further requirements from our research team, then please do not hesitate to ask, and we sincerely thank you for considering our revised manuscript.

Kind regards,

Callum Thomas.

Attachment Submitted filename: Response to Reviewers (7).docx

# Peer review: Decision Letter 1

**DOI:** 10.1371/journal.pone.0360525.r003

**Authors:** Kota V Ramana

28 Sep 2026

Blood Biomarkers of Long COVID: A Cross-Sectional Evaluation of Key Cytokines and the Coordination of Inflammation.

PONE-D-26-23542R1

Dear Dr. Faghy,

We’re pleased to inform you that your manuscript has been judged scientifically suitable for publication and will be formally accepted for publication once it meets all outstanding technical requirements.

Within one week, you’ll receive an e-mail detailing the required amendments. When these have been addressed, you’ll receive a formal acceptance letter and your manuscript will be scheduled for publication.

An invoice will be generated when your article is formally accepted. Please note, if your institution has a publishing partnership with PLOS and your article meets the relevant criteria, all or part of your publication costs will be covered. Please make sure your user information is up-to-date by logging into Editorial Manager at Editorial Manager® and clicking the ‘Update My Information' link at the top of the page. For questions related to billing, please contact billing support.

If your institution or institutions have a press office, please notify them about your upcoming paper to help maximize its impact. If they’ll be preparing press materials, please inform our press team as soon as possible -- no later than 48 hours after receiving the formal acceptance. Your manuscript will remain under strict press embargo until 2 pm Eastern Time on the date of publication. For more information, please contact onepress@plos.org.

Kind regards,

Kota V Ramana, Ph.D.

Academic Editor

PLOS One

Additional Editor Comments (optional):

Reviewers' comments:

Reviewer's Responses to Questions

**Comments to the Author**

Reviewer #1: All comments have been addressed

Reviewer #2: All comments have been addressed

**********

2. Is the manuscript technically sound, and do the data support the conclusions?

Reviewer #1: Yes

Reviewer #2: Yes

**********

3. Has the statistical analysis been performed appropriately and rigorously?

Reviewer #1: Yes

Reviewer #2: Yes

**********

4. Have the authors made all data underlying the findings in their manuscript fully available?

The PLOS Data policy

Reviewer #1: Yes

Reviewer #2: Yes

**********

5. Is the manuscript presented in an intelligible fashion and written in standard English?

Reviewer #1: Yes

Reviewer #2: Yes

**********

Reviewer #1: The authors have answered most of my previous comments. The manuscript is now clearer, and the conclusions are more careful.

They explained that this is an exploratory study, that no formal sample size calculation was done, and that multiple comparisons were adjusted. They also added limitations about comorbidities, COVID confirmation method, and missing cytokine values.

Some limitations still remain, including small subgroup sizes, cross-sectional design, and possible confounding from comorbidities. However, these are now described more clearly.

Overall, I think the revised manuscript is acceptable.

Reviewer #2: The manuscript has been carefully reviewed and is considered suitable for publication. The authors have adequately addressed the relevant issues, and no further substantive revisions are required at this stage.

Recommendation: Accept for publication.

**********

what does this mean?). If published, this will include your full peer review and any attached files.

If you choose “no”, your identity will remain anonymous but your review may still be made public.

**Do you want your identity to be public for this peer review?**  For information about this choice, including consent withdrawal, please see our Privacy Policy

Reviewer #1: No

Reviewer #2: **Yes:**  Fadrian

**********

# Editor report: Acceptance letter

**DOI:** 10.1371/journal.pone.0360525.r004

**Authors:** Kota V Ramana

PONE-D-26-23542R1

PLOS One

Dear Dr. Faghy,

I'm pleased to inform you that your manuscript has been deemed suitable for publication in PLOS One. Congratulations! Your manuscript is now being handed over to our production team.

At this stage, our production department will prepare your paper for publication. This includes ensuring the following:

* All references, tables, and figures are properly cited

* All relevant supporting information is included in the manuscript submission,

* There are no issues that prevent the paper from being properly typeset

You will receive further instructions from the production team, including instructions on how to review your proof when it is ready. Please keep in mind that we are working through a large volume of accepted articles, so please give us a few days to review your paper and let you know the next and final steps.

Lastly, if your institution or institutions have a press office, please let them know about your upcoming paper now to help maximize its impact. If they'll be preparing press materials, please inform our press team within the next 48 hours. Your manuscript will remain under strict press embargo until 2 pm Eastern Time on the date of publication. For more information, please contact onepress@plos.org.

You will receive an invoice from PLOS for your publication fee after your manuscript has reached the completed accept phase. If you receive an email requesting payment before acceptance or for any other service, this may be a phishing scheme. Learn how to identify phishing emails and protect your accounts at https://explore.plos.org/phishing.

If we can help with anything else, please email us at customercare@plos.org.

Thank you for submitting your work to PLOS One and supporting open access.

Kind regards,

PLOS One Editorial Office Staff

on behalf of

Dr. Kota V Ramana

Academic Editor

PLOS One
